# Expanding Research Capacity in Sub-Saharan Africa Through Informatics, Bioinformatics, and Data Science Training Programs in Mali

**DOI:** 10.3389/fgene.2019.00331

**Published:** 2019-04-12

**Authors:** Jeffrey G. Shaffer, Frances J. Mather, Mamadou Wele, Jian Li, Cheick Oumar Tangara, Yaya Kassogue, Sudesh K. Srivastav, Oumar Thiero, Mahamadou Diakite, Modibo Sangare, Djeneba Dabitao, Mahamoudou Toure, Abdoulaye A. Djimde, Sekou Traore, Brehima Diakite, Mamadou B. Coulibaly, Yaozhong Liu, Michelle Lacey, John J. Lefante, Ousmane Koita, John S. Schieffelin, Donald J. Krogstad, Seydou O. Doumbia

**Affiliations:** ^1^Department of Global Biostatistics and Data Science, Tulane University School of Public Health and Tropical Medicine, New Orleans, LA, United States; ^2^Faculty of Medicine and Odontostomatology, University of Sciences, Techniques and Technologies of Bamako, Bamako, Mali; ^3^Department of Mathematics, Tulane University, New Orleans, LA, United States; ^4^Sections of Pediatric & Adult Infectious Diseases, School of Medicine, Tulane University, New Orleans, LA, United States

**Keywords:** bioinformatics, data science, data capture and management systems, genetics, genomics, Human Heredity and Health in Africa (H3Africa), malaria, training

## Abstract

Bioinformatics and data science research have boundless potential across Africa due to its high levels of genetic diversity and disproportionate burden of infectious diseases, including malaria, tuberculosis, HIV and AIDS, Ebola virus disease, and Lassa fever. This work lays out an incremental approach for reaching underserved countries in bioinformatics and data science research through a progression of capacity building, training, and research efforts. Two global health informatics training programs sponsored by the Fogarty International Center (FIC) were carried out at the University of Sciences, Techniques and Technologies of Bamako, Mali (USTTB) between 1999 and 2011. Together with capacity building efforts through the West Africa International Centers of Excellence in Malaria Research (ICEMR), this progress laid the groundwork for a bioinformatics and data science training program launched at USTTB as part of the Human Heredity and Health in Africa (H3Africa) initiative. Prior to the global health informatics training, its trainees published first or second authorship and third or higher authorship manuscripts at rates of 0.40 and 0.10 per year, respectively. Following the training, these rates increased to 0.70 and 1.23 per year, respectively, which was a statistically significant increase (*p* < 0.001). The bioinformatics and data science training program at USTTB commenced in 2017 focusing on student, faculty, and curriculum tiers of enhancement. The program’s sustainable measures included institutional support for core elements, university tuition and fees, resource sharing and coordination with local research projects and companion training programs, increased student and faculty publication rates, and increased research proposal submissions. Challenges reliance of high-speed bandwidth availability on short-term funding, lack of a discounted software portal for basic software applications, protracted application processes for United States visas, lack of industry job positions, and low publication rates in the areas of bioinformatics and data science. Long-term, incremental processes are necessary for engaging historically underserved countries in bioinformatics and data science research. The multi-tiered enhancement approach laid out here provides a platform for generating bioinformatics and data science technicians, teachers, researchers, and program managers. Increased literature on bioinformatics and data science training approaches and progress is needed to provide a framework for establishing benchmarks on the topics.

## Introduction

African countries have long been disproportionately burdened by the “big three” infectious diseases (HIV and AIDS, tuberculosis, and malaria) and neglected emerging infectious diseases such as EVD and Lassa fever. African populations maintain the world’s highest levels of genetic diversity which decline proportionately with increasing distance from Africa (Tishkoff et al., 2009). Bioinformatics and data science [respectively, considered in this context as the methods and software tools for understanding biological data; and the unification of data design, collection and analysis (Hayashi, 1998; Wikipedia, 2019a)] research thrives on genetically diverse populations as population substructure variation contributes to the identification of true associations in complex disorders and drug response (Campbell and Tishkoff, 2008; Tishkoff et al., 2009; Quansah and McGregor, 2018). Research on these topics within Africa provide considerable opportunities for improving health outcomes through their application in infectious disease research, vaccine and drug development, and drug resistance patterns. The completion of the Human Genome Project and technological advances have led to significant cost reductions for genomic data acquisition and also provide immense opportunities for novel insights into etiology, diagnosis, and therapy (Tishkoff et al., 2009).

African researchers and participant populations have historically been underrepresented in GWAS. Through 2014, only 11 of the thousands of the GWAS have included African participants (Rotimi et al., 2014). While African countries such as South Africa have strong bioinformatics and data science capabilities, such capacity has been imbalanced across Africa, and many of its countries have yet to develop any of these capacities (Aron et al., 2017). Currently bioinformatics and data science degree programs are concentrated within several African institutions (Karikari et al., 2015b). Other factors negatively impacting bioinformatics and data science research in Africa include weak biomedical infrastructure; lack of governmental financial support; limited computational expertise; lack of participation in collaborative research beyond sample collection; and limited training opportunities, biorepositories, and databases (Tishkoff et al., 2009; Woolley et al., 2010; Rotimi et al., 2014; Karikari et al., 2015a; World Health Organization, 2015; Nielsen et al., 2017).

To fully benefit from advances in bioinformatics and data science research, it is imperative to train the next generation of African scientists on their use (Adoga et al., 2014; Human Heredity and Health in Africa, 2018). Tastan Bishop et al. (2015) note that the shortage of trained bioinformaticians is among the main obstacles in the development of bioinformatics in Africa (Tastan Bishop et al., 2015). Doumbo and Krogstad (1998) note that doctoral training on advanced topics are essential for African countries to define and implement their own health priorities (Doumbo and Krogstad, 1998). These demands call for building local university programs and infrastructure for establishing environments that are conducive for bioinformatics and data science training. Bioinformatics is known to require less infrastructural investments than other bench science initiatives, but essential resources are necessary such as powerful computer systems, reliable high-speed internet, access to databases and software programs, and reliable electricity (Karikari et al., 2015a). Karikari et al. (2015a) also note the importance of research infrastructure, research funding, training programs, scientific networking, and collaborations as key elements for developing bioinformatics expertise (Karikari et al., 2015a). Other factors affecting the implementation of training programs include teaching laboratories, server systems, airfare cost, timeliness of visas, suitable computational infrastructure, socio-political stability, and availability of open training spots (Aron et al., 2017; Shaffer et al., 2018). This capacity may be gained through research and training on overlapping computationally intensive topics such as data management and data capture (Shaffer et al., 2018). Attwood et al. (2017) describe the importance of data management, data storage, data integration, and data sharing, and data science in bioinformatics training (Attwood et al., 2017). The importance of DCMSs is regularly noted in the literature as a key tool for establishing sustainable and collaborative research efforts (Lansang and Dennis, 2004; World Health Organization, 2004; Abou Zahr and Boerma, 2005; Kirigia and Wambebe, 2006; Gezmu et al., 2011; Gutierrez et al., 2015; Mulder et al., 2017; Shaffer et al., 2018).

Multi-country organizations such as the H3Africa and H3Africa BioNet (H3ABioNet) consortiums have yielded extensive training and research opportunities within Africa (Human Heredity and Health in Africa, 2013; National Institutes of Health, 2018). The H3Africa initiative aims to study genomics and environmental diseases to improve the health of African populations, partnering between the AESA, the Wellcome Trust, the ASHG, and the NIH (Adoga et al., 2014; Human Heredity and Health in Africa, 2018). The H3Africa Consortium had the effect of diversifying the bioinformatics skills and training in Africa, providing genomics training for over 500 Africans approximately 5 years (Mulder et al., 2018a). H3ABioNet is a Pan-African bioinformatics network consisting of 32 bioinformatics research groups in 15 African countries and partner institutions in the United States providing bioinformatics training in both introductory bioinformatics topics and specialized topics such as next generation sequencing (NGS) and GWAS (National Institutes of Health, 2018). The H3ABioNet bioinformatics training platform includes distance-based online training courses using virtual classrooms across 20 African institutions (Gurwitz et al., 2017). The Eastern Africa Network of Bioinformatics Training (EANBitT) provides bioinformatics training in Kenya as part of a M.Sc. program in bioinformatics (International Centre of Insect Physiology and Ecology, 2018). Doctoral training in bioinformatics is also provided in Botswana and Uganda through the Collaborative African Genomics Network [CAfGEN; (Mlotshwa et al., 2017)]. Karikari (2015) discuss the current bioinformatics training programs in Ghana (Karikari, 2015). Tastan Bishop et al. (2015) lay out the development of bioinformatics as a discipline and list the current bioinformatics degree programs in Africa (Tastan Bishop et al., 2015). Mulder et al. (2018b) provide guidelines for competencies for bioinformatics training in Africa. The African Genomic Center maintains the first genome sequencing facility that was launched in Cape Town in 2018 and includes a strong bioinformatics training component (SAMRC, 2018). Other organizations promoting bioinformatics in Africa include The African Society for Bioinformatics and Computational Biology and formerly The ABioNET (SciDevNet, 2004; African Society for Bioinformatics and Computational Biology, 2019).

The focus of the current work are bioinformatics and data science training in sub-Saharan Mali. Research in Mali has emphasized malaria as it is the country’s primary cause of morbidity and mortality, representing 42% of consultations in its health centers (Sissoko et al., 2017). Malaria control strategies in Mali have emphasized universal intervention coverage, epidemic and entomological surveillance, and targeted operational research (President’s Malaria Initiative, 2018). Substantial progress in malaria reduction has occurred through scaling up malaria prevention and control interventions resulting in a nearly 50% reduction in malaria mortality rates in children under 5 years of age (President’s Malaria Initiative, 2018). However, drug resistance to antimalarial drugs have complicated efforts to fully control malaria. The utilization of genomic and clinical data to understand parasite evolution, predict behaviors of resistance to new antimalarial medication, and inform strategies to prevent the spread of drug-resistant malaria is thus of great importance (Flegg et al., 2011; Fairhurst et al., 2012; Maiga et al., 2012; Takala-Harrison and Laufer, 2015; Oboh et al., 2018). Other infectious diseases with significant burden in Mali include leishmaniasis, filariasis, and tick- borne diseases. Neglected infectious diseases that have not been extensively studied (but not necessarily absent) in Mali include Lassa fever and EVD (Schieffelin et al., 2014; Shaffer et al., 2014; Traore et al., 2016).

As with many countries in sub-Saharan Africa, Mali has significant limitations in developing, implementing, sustaining, and expanding innovative mechanisms for research efforts and clinical trials that are central to its health improvement (Miiro et al., 2013; Mwangoka et al., 2013; Richie et al., 2015; Dicko et al., 2016; Niare et al., 2016). Recent studies on health information systems (HIS) in Mali reported limited expertise in data management, data analysis, and report generation (MEASURE Evaluation, 2014, 2016). Mali also shares the difficult task of collecting data through a weak HIS for monitoring the health of its population (Asangansi, 2012; Ndabarora et al., 2014). Despite these limitations, research investments in Mali have been substantial. Mali was established as an International Center of Excellence in Research (ICER) in 2002 and is currently ranked as the seventh highest investment country for malaria research (Head et al., 2017). The USTTB regularly serves as the lead institution research and training projects, including several recent awards as part of the H3Africa initiative (Landoure et al., 2016; Human Heredity and Health in Africa, 2019).

Here we describe an incremental approach for engaging the next generation of African scientists in research through a progressive sequence of informatics, bioinformatics, and data science training programs at the USTTB. We describe the approaches, developments, and challenges incurred culminating with the West African Center of Excellence for Global Health Bioinformatics Research Training program in an effort assist researchers for reaching underserved populations in similar environments.

## Materials and Methods

### Study Site

Situated in urban Bamako, Mali, USTTB is comprised of schools of medicine, pharmacy, and basic sciences; an institute of applied science; and research laboratories focusing on malaria, tuberculosis, and retrovirology (Harvard T.H. Chan School of Public Health, 2018). The site maintains teaching computer laboratories; server systems; and a formal data center including computer workstations, printers and internet access in controlled-access spaces. USTTB is a member of the REDCap (Vanderbilt, TN) Consortium. The site is situated near the epicenter for a host of infectious diseases and is surrounded by numerous complementary research efforts and networks, including the West Africa International Centers of Excellence for Malaria Research [ICEMR (National Institute of Allergy and Infectious Diseases, 2018a)].

### An Incremental Approach for Engaging Underserved Populations in Bioinformatics and Data Science Research

Formal research and training infrastructure at USTTB dates back to 1989 with the launch the MRTC. The facility maintained hardwired internet access, laboratories, classrooms, conference rooms, and a library (Science Blog, 2000). The MRTC supported a host of internationally funded research projects (particularly the NIAID) and training programs and worked closely with Mali’s National Malaria Control Program (NMCP; Science Blog, 2000). While the MRTC’s mission was not initially focused on molecular research, it spawned growth in the area through its capacity building, particularly in the area of epidemiology. Bioinformatics was formally introduced to Mali in 2003 through the African Center for Training in Functional Genomics of Insect Vectors of Human Disease (AFRO VECTGEN), which was sponsored by the WHO as part of its Special Programme for Research and Training in Tropical Diseases (TDR) initiative. A timeline of incremental developments in bioinformatics and data science capacity building, research and training at USTTB are listed in Table 1.

**Table 1:** Timeline of key milestones in bioinformatics and data science at USTTB culminating with a bioinformatics and data science training program.

Year	Key development in bioinformatics and data science capacity building, training, or research	Sponsor
1989	Malaria Research and Training Center (MRTC)	NIH/NIAID/National School of Medicine and Pharmacy of Bamako
1999	International Training in Medical Informatics (ITMI) program	NIH/FIC
2002	International Centers for Excellence in Research (ICER)	NIH/NIAID
2003	African Center for Training in Functional Genomics of Insect Vectors of Human Disease (AFRO VECTGEN)	WHO/TDR
2004	Informatics Training in Global Health (ITGH); Mali Service Center	NIH/FIC/NIAID
2004–2007	Short term training for bioinformatics and functional genomics (USTTB hosted 1 of 5 such training centers)	WHO
2010	West Africa ICEMR	NIH/NIAID
2013	Funded as lead institution for H3Africa initiative	NIH/Wellcome Trust
2015	Master of science in bioinformatics program	NIH/H3ABioNet/H3Africa NIH/NIAID/BioTeam/EMC
2016	African Center of Excellence in Bioinformatics (ACE)	Corporation/Hewlett Packard Corporation/Intel Corporation Health and Life Sciences Group
2017	West African Center of Excellence for Global Health Bioinformatics Research Training program	NIH/FIC/H3Africa

*FIC, Fogarty International Center; H3Africa, Human Heredity and Health in Africa; ICEMR, West Africa International Centers of Excellence for Malaria Research; NIAID, National Institute of Allergy and Infectious Diseases; NIH, National Institutes of Health; TDR, Special Program for Research and Training in Tropical Diseases; USTTB, University of Sciences, Techniques and Technologies of Bamako, Mali; WHO, World Health Organization*.

The West African Center of Excellence for Global Health Bioinformatics Research Training program was launched in 2017 (Africa). The program leveraged infrastructure and personnel from: two earlier informatics training programs, a malaria research project, the USTTB bioinformatics M.Sc. program, and the African Center of Excellence in Bioinformatics (ACE) teaching computer laboratories (Doumbia et al., 2012; Koita et al., 2016, 2017; National Institute of Allergy and Infectious Diseases, 2018b). Descriptions of these efforts follow.

### International Training in Medical Informatics (ITMI)

From 1999 to 2003, the ITMI program provided short and long term training in informatics for Malian researchers at the MRTC and governmental health agencies across West Africa. The ITMI program was complemented with research on determinants of drug resistance, immune evasion and virulence in malaria, development of field research sites to study drug resistance, human response to malaria, pathogenesis of severe malaria, and malaria vaccine trials. The informatics focus for the ITMI occurred in the sense of research question formulation and data collection, capture, linkage processes, management, and analysis. The program included five trainees with the overall goal of completing master’s degrees in public health and preparing manuscripts and submitting them for publication in peer-reviewed journals.

### Informatics Training in Global Health (ITGH)

Building on the ITMI program, the ITGH program was a carried between 2004 and 2011 and provided training toward completion of M.Sc. degrees in public health. Several trainees also participated in an online master’s diploma program known as epidemiology and public health (Epidemiologie et Sante Publique en ligne; ESPEL). With training delivered entirely in French, ESPEL was a consortium serving Francophone countries in the Mediterranean and North Africa through the University of Bordeaux with courses in statistics and epidemiology. Course instruction in the ESPEL program was provided by USTTB medical faculty and online tutoring tools.

### West Africa International Centers of Excellence for Malaria Research (ICEMR)

The International Centers of Excellence for Malaria Research is a network of research centers with a common mission to eradicate and control malaria in Asia, Africa, Latin America, and the Southwest Pacific (National Institute of Allergy and Infectious Diseases, 2018a). Between 2010 and 2017, the West African ICEMR network carried out longitudinal malaria studies at four sites in Senegal, The Gambia, and Mali (Doumbia et al., 2012). These countries provided four study sites with differential seasonal prevalence of *Plasmodium falciparum* (*P. falciparum*) infection and incidence in uncomplicated malaria (Shaffer et al., 2018). The primary goal of the study was to collect epidemiologic, clinical, and molecular data to better understand the transmission and human impact of malaria. Significant byproducts of this work were trained research personnel and established DCMS (Shaffer et al., 2018). These efforts continued in 2017 focusing on the study of malaria control interventions and antimalarial drug resistance (National Institute of Allergy and Infectious Diseases, 2018b).

### African Centers of Excellence in Bioinformatics Program (ACE)

The ACE program is a public-private partnership with the NIAID and the FNIH to strengthen bioinformatics research capacity in low and middle income (LMIC) African countries (Foundations for the National Institutes of Health, 2018). The ACE program was launched at USTTB in 2016 and included a teaching computer laboratory and e-classroom with Adobe Connect (San Jose, CA, United States) capacity for real time learning and instruction.

### Africa Center for Training in Functional Genomics of Insect Vectors of Human Disease (AFRO VECTGEN)

The AFRO VECTGEN program was initiated in 2003 through a partnership with the WHO Special Programme for Research and Training in Tropical Diseases (TDR) and the MRTC Department of Medical Entomology and Vector Ecology (Doumbia et al., 2007). The program provided training for African scientists on genome sequence data management and analysis and functional genomics for research on vector-borne diseases.

### USTTB Master of Science (M.Sc.) Bioinformatics Program

USTTB maintains a M.Sc. in Bioinformatics program established in 2015 in collaboration with the H3ABionNet Consortium. The program is one of only 13 such programs in 7 African countries (Tastan Bishop et al., 2015; Mulder et al., 2016). The program includes 20 courses arranged over 4 semesters with cohorts of 15 students over quarterly semesters, including three semesters of coursework and short-term internships and a single semester of thesis research in bioinformatics. The 1st year of study includes two semesters of core coursework equivalent to 60 academic credits (European Credit system), and the 2nd year consists of 60 credits of coursework and a 4-month practicum and a master thesis research project. Training is provided in collaboration by USTTB faculty; the H3ABioNet Consortium (from instructors in Tunisia, South Africa, and Ghana); and collaborating institutions in France and the United States (through video conferencing and webinars). The program’s curriculum is shown in Table 2.

**Table 2:** USTTB Master of Science (M.Sc.) in Bioinformatics program curriculum.

Semester^1^	Code	Course title	Credits
1	BIN101	Basic Mathematics	4
	BIN102	Basic Structural Biology	5
	BIN103	Biostatistics I	5
	BIN104	English for Scientists	3
	BIN105	Introduction to Programming	4
	BIN106	Introduction to Linux and Shell Scripting	4
	BIN107	Cellular and Molecular Biology	5
2	BIN201	Genomics and Proteomics	6
	BIN202	NGS and Data Analysis	6
	BIN203	Evolution and Phylogeny	5
	BIN204	Web Programming	4
	BIN204	Molecular Modeling	4
	BIN205	Programming for Bioinformatics	5
3	BIN301	Metabolomics and *In silico* Analysis	5
	BIN302	Research Methodology and Ethics	5
	BIN303	Bioinformatics Tools for Public Health	3
	BIN304	Databases	4
	BIN305	Biostatistics II	4
	BIN306	Scientific Writing and Communication	3
	BIN307	Project Management	3
	BIN308	Population Genetics and GWAS	3
4	BIN400	Master’s thesis research project	30
			Total: 120

*^*1*^Semesters based on quarterly time periods.*

A key component of the curriculum for engaging underserved populations in research included a formal course on English speaking and writing in scientific research (Table 1, course code BIN 103).

### West African Center of Excellence for Global Health Bioinformatics Research Training

Launched in October 2017, the West African Center of Excellence for Global Health Bioinformatics Research Training is a collaborative bioinformatics data science and training program between USTTB and Tulane University. The program provides bioinformatics and data science training to faculty and students at USTTB and is sponsored by the NIH Fogarty International Center as part of the H3Africa initiative. The program seeks to establish a sustainable bioinformatics and data science research training program at USTTB, focusing on advancing the USTTB bioinformatics curriculum, increasing faculty and student authorship in bioinformatics and data science journals, grant proposal development, and improving success in gaining extramural research funding.

## Results

The primary outcomes of the ITMI and ITGH training programs included numbers of college degrees earned and publication frequencies and rates. These programs laid the foundation for subsequent research and training efforts. Among the trainees in the ITMI and ITGH programs were investigators the West Africa ICEMR and West African Center of Excellence for Global Health Bioinformatics Research Training program.

### International Training in Medical Informatics (ITMI)

This program provided long-term training to five trainees between 1999 and 2003. Each of these trainees successfully completed a M.Sc. in Public Health (MSPH) degree. The impact of the ITMI on publication productivity is shown in Table 3.

**Table 3:** Publication productivity for *n* = 5 trainees enrolled in the International Training in Medical Informatics program (ITMI).

Publication type	Pre-training (2000–2003)	Post-training (2004–2017)	% Increase in publications per year	*P*-value^1^
First or second authorship	2 (0.10)	55 (0.79)	690%	<0.001
Third or higher authorship	8 (0.40)	99 (1.41)	253%	<0.001

*Results in second and third columns expressed as frequency of publications and average publications per trainee per year. ^*1*^Comparison of person-time publication rates between pre- and post-training time periods*.

Publication rates per year in first authorship and third and higher authorship increased by 690% (0.10 per year to 0.79 per year) and 253% (0.40 per year to 1.41 per year), respectively, following the ITMI training program. Each of these increases was statistically significant (*p* < 0.001). Publications following the ITMI program were focused in the areas of malaria interventions, vaccine development, and epidemiology (Sagara et al., 2014; Portugal et al., 2017).

### Informatics Training in Global Health (ITGH)

The ITGH program provided short and long term informatics training in Mali between 2004 and 2011. The program included 53 short-term trainees and 7 long-term trainees from the MRTC, local governmental agencies, field sites and neighboring Francophone West African countries. Short term workshop training was delivered in both French and English, and ten short-term trainees completed the ESPEL online diploma training in biostatistics and epidemiology. Three of the long-term trainees earned master of public health degrees in biostatistics programs, and four of the long-term trainees completed the online ESPEL training program.

### West African Center of Excellence for Global Health Bioinformatics Research Training Program (WABT)

**FIGURE 1:**
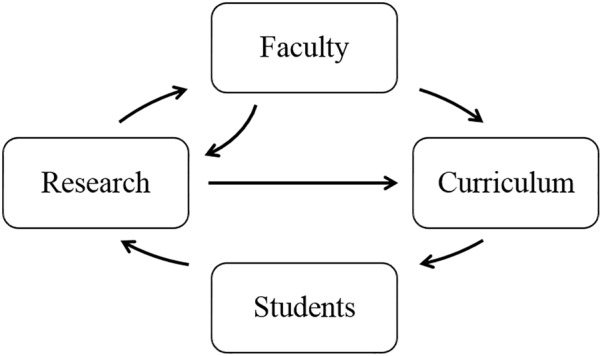
Feedback loop for bioinformatics and data science training program components in the West African Center of Excellence for Global Health Bioinformatics Research Training program.

The WABT was sponsored by the National Institutes of Health through its H3Africa initiative. The USTTB served as the WABT’s lead institution, partnering with Tulane University (New Orleans, LA, United States) and the University of Strasbourg (Alsace, France). The WABT integrated three intertwined training components, namely faculty training and development, curriculum enhancement, and student training enhancement for students enrolled in the USTTB master’s degree program in bioinformatics. The feedback loop illustrating the approach for launching new trainees into academic and research positions is shown in Figure 1.

The program provided a direct pipeline of trainees into the USTTB bioinformatics program and local research projects. An advisory board provided independent oversight and insight for the program. The three tiers of enhancement covered in the training program follow.

#### Faculty Enhancement

Faculty trainees were recruited from USTTB faculty with responsibilities in developing and overseeing the USTTB bioinformatics and data science curriculum. This component was carried out through participation in scientific workshops; delivery of oral and poster presentations at scientific conferences; mentorship on proposal development and manuscript preparation; and development and implementation of an annual bioinformatics symposium at the USTTB site. Research proposal topics focused on mobile health in malaria surveillance and efficacy evaluation for seasonal malaria chemotherapy in malaria prevention through the application of bioinformatics and data science approaches and technologies.

#### Curriculum Enhancement

Training activities included mentored program and curriculum development for the USTTB M.Sc. in bioinformatics program. The program’s course competencies were compared and evaluated according to Mulder et al. (2018a,b). Curriculum modifications included an expansion of the program’s component through providing options for completing thesis work at outside institutions. Additionally, a certificate training program in bioinformatics was developed based on current course offerings to expand participation and generate additional revenue for supporting the program. The ultimate goal of the curriculum enhancement activities was to lay the groundwork for a doctoral program in bioinformatics at USTTB.

#### Student Enhancement

**FIGURE 2:**
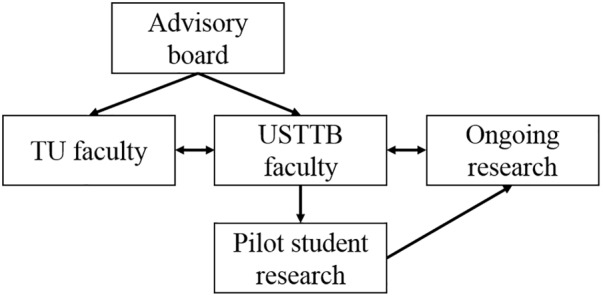
Evaluation and administration of student pilot research projects in the West African Center of Excellence for Global Health Bioinformatics Research Training program. TU, Tulane University.

The project provided partial scholarships for current and incoming students in the USTTB M.Sc. in bioinformatics program as well as partial scholarships for related doctoral programs. Trainees were recruited among students enrolled in the USTTB M.Sc. in bioinformatics program or doctoral students working in research programs with bioinformatics focuses. Training activities included “study abroad” training at outside institutions through the following mentored activities: formal coursework; literature review preparation; data capture, management, and analysis; and manuscript preparation. Manuscript data were provided through the West Africa ICEMR research projects. Trainees were responsible for attending and presenting research findings at professional research conferences, including the American Society of Tropical Medicine and Hygiene (ASTMH) and H3Africa consortium meetings. Funds were allocated for pilot research projects per year in the amount of $10,000 USD, which were intended to foster mentorship, incorporation of research into the classroom, and research evaluation. An online portal was developed for proposal submissions, and proposals were reviewed and scored by the training program’s key investigators and advisory board (Figure 2).

#### Workshop Training

Workshop training was provided annually at USTTB aimed toward two aspects: (1) enabling junior trainees to effectively manage and interpret genetic data, including major bioinformatics database sources and integration with biological data; and (2) performing computational tasks and carrying out analytical approaches to process, analyze, and interpret biological data. The program’s workshop themes are listed in Table 4.

**Table 4:** Training workshop sequence for the West African Center of Excellence for Global Health Bioinformatics Training program.

Workshop themes	Applications used	Workshop goals
Introductory biological concepts for bioinformatics; Data management and visualization	*R; Bioinformatics databases*	Manage data; Effectively use bioinformatics databases
Bioinformatics software applications in malaria research; Introductory statistical genetics; introductory disease mapping	*R; Bioinformatics databases; ArcGIS; ArcGIS Online*	Perform bioinformatics analyses
Development and implementation of REDCap databases; Intermediate statistical genetics	*REDCap; R; Python*	Carry out data capture and management; Utilize statistical genetics applications
Intermediate disease mapping; Advanced statistical genetics	*ArcGIS; ArcGIS Online; R; Python*	Generate disease maps; Implement advanced statistical genetics approaches
Manuscript preparation; Data management plan preparation for bioinformatics research	*R; Python; Microsoft Office*	Generate tables, graphs, and draft manuscripts; Present research findings in written and oral discourses

The official language in Mali is French, but Bombari is the most widely spoken (Wikipedia, 2019b). The workshop training was delivered in English, and periodic translation summaries in French were delivered by USTTB faculty. Each day of the workshops concluded with student oral summaries of concepts and activities. Certificates of completion were awarded following successful completion of the workshops and were presented by the USTTB president and the project’s principal investigators.

#### Additional Training Activities

Financial program management training was provided for USTTB financial administrators through in-person discussion sessions with trained sponsored projects personnel. Training was also provided on biographical sketch development, COI and disclosure, and research ethics.

### Challenges

The challenges incurred during the bioinformatics and data science training program included language barriers, complexity in obtaining United States. Exchange Visitor visas (J-1), high-speed internet availability, and the lack of discounted software portals. While the training was primarily delivered in English, many of the trainees were not fully fluent in English. Also, this effort required the availability of high-speed bandwidth for utilizing software extensions and accessing biomedical databases. While high-speed bandwidth was available for training at USTTB, its funding was dependent on ongoing short-term funding. The lack of a discounted software portal for commercial software presented challenges for acquiring and upgrading several common software applications such as Microsoft Access (Redmond, WA, United States). This effort focused on freeware applications including R, REDCap, ArcGIS Online (Esri, Redlands, CA, United States), and QGIS (formerly Quantum GIS; Open Source Geospatial Foundation, Chicago, IL, United States). While the training program included cohorts of trainees with similar academic focuses, the participants with bioinformatics expertise ranged from beginning to advanced skill sets. The program strategy here incorporated basic biological concepts prior to covering more advanced topics in bioinformatics and data science. The lack of industry opportunities for trainees primarily limited post-training employment prospects to academia, research, and governmental health agencies.

**FIGURE 3:**
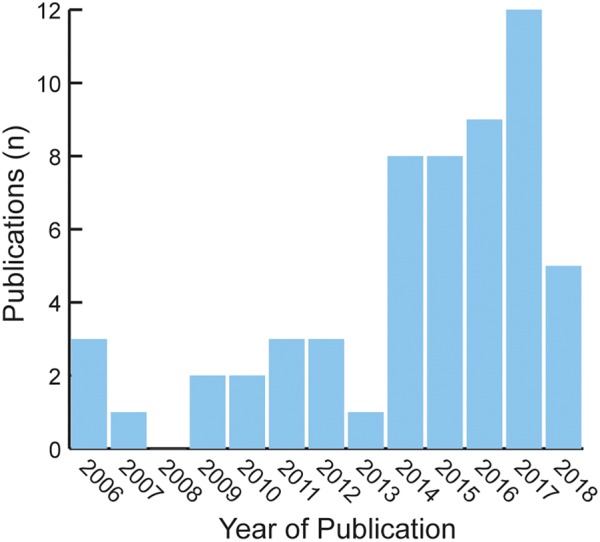
Publication hits using the PubMed biomedical database search engine including key words *Mali bioinformatics*.

Using a PubMed search with key words *Mali bioinformatics* yielded *N* = 63 publications between 2006 (the year of the first observed bioinformatics publication) and November 2018. None of these hits focused exclusively on bioinformatics or data science training (Figure 3).

An analog search for the Netherlands {with a population of approximately 17,084,719 [2017 estimate], slightly smaller than Mali’s total population of 17,885,245 [2017 estimate]; (United States Central Intelligence Agency, 2018)} yielded 8,027 hits. These results illustrate that publication in bioinformatics remains extremely weak in Mali relative to developed countries with similar populations.

### Sustainability

Sustainability was a core part of the program’s study design that was envisioned through its development in a university setting capable of: maintaining key resources during lapses in short-term funding, generating tuition and fees, developing workforces and human capital, and providing teaching computer and wet laboratory capacity. The program’s sustainability approaches and measures are shown in Table 5.

**Table 5:** Sustainable measures for the West African Center of Excellence for Global Health Bioinformatics Research Training program.

Program focus	Sustainable measure
University setting	Institutional support for core elements
Bioinformatics courses	University tuition
Bioinformatics certificate program	University fees
Short-term research projects	Resource sharing
Training offered through companion training programs	Resource sharing
Collaborative grant proposals and research	Elevated university ranking
H3Africa platform	Elevated student and faculty visibility and more extensive research networks
Workforce training	Increased research proposals and industry involvement
Pilot funding for student research projects	Increased independent research

## Discussion

Bioinformatics and data science expertise has arguably the most potential and impact in underserved parts of Africa due its high levels of disease and genetic diversity. The computational capacity and dynamic nature of bioinformatics research and training necessitate incremental processes in capacity building and training on related data intensive topics such as data capture and management. This capacity may be used to establish research networks and improve site suitability for hosting additional research. The training programs here yielded a sustainable platform for launching trainees into academia and complimentary research projects. The training programs here benefited from a longstanding partnership between USTTB and Tulane University in both training and research capacities. This partnership fostered local participation in content and program development to target the specific needs and health outcomes found in Mali. The authors here acknowledge that the progress described throughout this work did not operate in a vacuum and directly benefitted from a host of efforts by other researchers over the past several decades. Indeed the teaching laboratory facilities available through the African Center of Excellence in Bioinformatics and the bioinformatics curriculum development by Mulder et al. (2016) were vital to the launch of our bioinformatics and data science training program.

We found English training to be a key component for engaging underserved populations, and thus our earlier informatics training programs included formal English training. We believe that providing English training at outside institutions is becoming more difficult as United States visa programs such as the Visitor Exchange (J-1) visa program mandate English proficiency (U.S. Department of State, 2019). Inclusion of formal coursework in English in the USTTB bioinformatics curriculum also provided key advancements in this regard. It is of central importance for African universities with research missions to incorporate research-driven and overlapping computationally intensive research topics courses into their curriculum, including biostatistics, GISs, bioinformatics, and data science to foster a workforce capable of competing for large-scale research projects.

### Trainee Outcomes

Trainee outcomes were considered primarily in terms of publications, grant proposal submissions and awards, research conference presentations, and employment outcomes. In the absence of strong industry or pharmaceutical presence, it is likely that trainees will ultimately gain employment in research, academic, or government settings. Emphasizing proposal preparation within the training programs therefore has great utility in this regard. The training efforts in this work benefitted from several complementary malaria research projects that provided training and data sources for manuscript development and publication. Our programs also benefitted from the biannual H3Africa research conferences, which provided an international venue for our trainees to present their work in oral or written discourses. Professional responsibilities in Africa are perhaps less dependent on scientific publishing for measuring scientific productivity than in other parts of the world, and thus additional incentives for publishing may be useful to conform to the extramural funding process where publication is highly prioritized. One such incentive occurs at the University of Cape Town where governmental supplements are provided for completed publications (Whitworth et al., 2010). While overall publication rates are improving across African institutions, they are not always available through search engines such as PubMed. Schoonbaert (2009) notes that a more complete resource for African literature is CABI’s Global Health Database (Schoonbaert, 2009). Similar issues may arise for country-specific grants or foundation grants as they often go uncaptured in research repositories such as NIH RePORTER (U.S. Department of Health and Human Services, 2018). To this end, country research databases may be useful in improving research visibility.

### Correlate Training

Ideally trainees should develop diverse research portfolios including topics focused on practical needs with utility in all facets of research, such as DCMS development and oversight. These practical skills provide opportunities over the entire course of research endeavors as opposed to sole skill sets in advanced analytical techniques that are suited for latter stages research. The challenges associated with bioinformatics and data science training parallel those for related data intensive research processes such as DCMS. Core bioinformatics and data science research infrastructure shares common elements with data intensive epidemiological and clinical research such as the setup of data systems, data management, and data warehousing. Because of the overlap in bioinformatics and DCMS responsibilities, we provided training on the development and use of REDCap databases and tablet-based data collection. Other practical training in our programs included biographic sketch and curriculum vitae and resume development and program management.

### Regional Thinking and Sustainability of Bioinformatics and Data Science Training Programs

While the training programs covered in this work focused on Mali, we believe that they made a positive impact more broadly across the region of sub-Saharan Africa. These programs regularly hosted trainees from Mali’s neighboring countries, including Nigeria and Ghana. The West Africa ICEMR also fostered collaborations among multiple sub-Saharan countries, including Mali, Senegal, and The Gambia.

Regional training approaches may increase research participation for countries lacking necessary capacity for hosting training efforts independently. The utility of regional-based approaches is recognized to yield sharing of study protocols and standardization of case definitions and reporting practices (Shaffer et al., 2018). Integrating data sources across study sites or countries provides opportunities for more advanced, multifactor approaches for evaluating treatments and vaccines. Such efforts are facilitated when host countries consider the health problems of neighboring countries as their own. Defining appropriate regional groupings may also consider the absence of disease as viable opportunities for control populations in research. Virtual regional infrastructures have also been shown to improve engagement with countries with sparse research resources (Jennings et al., 2004).

Long-term sustainability of training and capacity development in Africa will likely require additional support within the host countries in the area of research and development. Among 13 African countries (Cameroon, Gabon, Ghana, Kenya, Malawi, Mali, Mozambique, Nigeria, Sengal, South Africa, Tanzania, Uganda, and Zambia), only 3 countries (Uganda, Malawi, and South Africa) achieved a modest goal for spending at least 1% of GDP on research and development. GDP expenditures on research and development among the remainder of surveyed countries ranged between 0.20 and 0.48% (NEPAD Planning and Coordinating Agency, 2010). By contrast, these expenditures for the United States and Japan were 2.74 and 3.15, respectively (The World Bank, 2019).

### Clinical Trials Infrastructure and Drug Development

Increased pharmaceutical involvement within Africa is greatly needed for developing bioinformatics and data science expertise and clinical research participation within the continent. Koita et al. (2016) note that key priorities in West Africa are the development of clinical research facilities and the training of host country investigators to ensure that the facilities and expertise necessary to evaluate candidate interventions are available in endemic regions when and where they are needed (Koita et al., 2016). The authors also note that many treatments deployed in Africa may have never included participants in their target countries. Bioinformatics and data science training programs provide an opportunity for showcasing workforce capacity to attract pharmaceutical and commercial investment. In turn, such investments will likely provide competitive advantages for short-term research. Partnerships with pharmaceutical companies may also serve as another means for sustaining core infrastructure during lapses in short-term funding.

### Importance of Literature on Bioinformatics and Data Science Training Efforts

To our knowledge, this is the first manuscript on bioinformatics and data science training in Mali. Additional literature on bioinformatics and data science training in Africa is needed for establishing training priorities, monitoring progress, and developing goal-based strategies for its improvement. Such literature also allows investigators developing new training programs to build on prior efforts and adapt training approaches. It is therefore essential for journal publishers to recognize the importance of publishing work on training programs as they often serve as the backbone for their associated research.

## Conclusion

Bioinformatics and data science training programs in developing countries necessitate incremental and collaborative strategies for their feasible and sustainable development. The progress described here covered decades of collaborative efforts centered on training and research on computationally intensive topics. These efforts laid the groundwork and platforms conducive for hosting a bioinformatics and data science training program in Mali. Training programs are perhaps best facilitated through Africa’s university systems as they are perhaps best positioned to maintain core resources during lapses in short-term funding. While bioinformatics and data science training programs are rapidly growing across Africa, much of the continent currently lacks substantial commercial investment and is reliant on short-term funding for training and research efforts. It is therefore critical to incentivize, commercial and governmental investment within African countries to complement short-term funding efforts. It is also of central importance to publish literature on scientific training programs to monitor and evaluate progress, develop standards, and share training approaches and experiences.

## Author Contributions

JGS conceived and drafted the manuscript which was reviewed and approved by all authors. JGS, FM, MW, CT, JL, OT, SKS, DK, and SD participated in study design. JGS, FM, JL, SKS, OT, MW, DK, JJL, MD, MS, DD, MT, YK, AAD, ST, BD, MC, OK, YL, and SD assisted in carrying out the training program. JSS and ML assisted in manuscript editing and consultation.

## Conflict of Interest Statement

The authors declare that the research was conducted in the absence of any commercial or financial relationships that could be construed as a potential conflict of interest.

## Abbreviations

ABioNET: African Bioinformatics Network
ACE: African Centers of Excellence in Bioinformatics program
AESA: Alliance for Accelerating Science in Africa
ASHG: African Society of Human Genetics
COI: conflict of interest
DCMS: data capture and management systems
EVD: Ebola virus disease
FNIH: Foundations for the National Institutes of Health
GDP: Gross domestic product
GIS: geographic information systems
GWAS: Genome-wide association studies
H3Africa: Human Heredity and Health in Africa
ICEMR: West Africa International Centers of Excellence for Malaria Research
IRB: Institutional review board
ITGH: Informatics Training in Global Health
ITMI: International Training in Medical Informatics
LMIC: Low and middle income countries
M.Sc.: Master of Science
MRTC: Malaria Research and Training Center
MSPH: Master of Science in Public Health
NGS: next generation DNA sequencing
NIAID: National Institute of Allergy and Infectious Diseases
NIH: National Institutes of Health
NMCP: Mali National Malaria Control Program
USTTB: University of Sciences, Techniques and Technologies of Bamako, Mali
WHO: World Health Organization

## References

[B1] Abou Zahr C. Boerma T. (2005). Health information systems: the foundations of public health. *Bull. World Health Organ.* 83 578–583. 16184276 PMC2626318

[B2] Adoga M. P. Fatumo S. A. Agwale S. M. (2014). H3Africa: a tipping point for a revolution in bioinformatics, genomics and health research in Africa. *Source Code Biol. Med.* 9:10. 10.1186/1751-0473-9-10 24829612 PMC4019950

[B3] African Society for Bioinformatics and Computational Biology (2019). *African Society for Bioinformatics and Computational Biology [Online].* Available at: http://www.asbcb.org/ (accessed February 25 2019).

[B4] Aron S. Gurwitz K. Panji S. Mulder N. (2017). H3ABioNet: developing sustainable bioinformatics capacity in Africa. *EMBnet J.* 23:e886 10.14806/ej.23.0.886

[B5] Asangansi I. (2012). Understanding HMIS implementation in a developing country ministry of health context - an institutional logics perspective. *Online J. Public Health Inform.* 4:ojphi.v4i3.4302. 10.5210/ojphi.v4i3.4302 23569646 PMC3615828

[B6] Attwood T. K. Blackford S. Brazas M. D. Davies A. Schneider M. V. (2017). A global perspective on evolving bioinformatics and data science training needs. *Brief Bioinform.* 20 398–404. 10.1093/bib/bbx100 28968751 PMC6433731

[B7] Campbell M. C. Tishkoff S. A. (2008). African genetic diversity: implications for human demographic history, modern human origins, and complex disease mapping. *Annu. Rev. Genomics Hum. Genet.* 9 403–433. 10.1146/annurev.genom.9.081307.164258 18593304 PMC2953791

[B8] Dicko A. Brown J. M. Diawara H. Baber I. Mahamar A. Soumare H. M. (2016). Primaquine to reduce transmission of Plasmodium falciparum malaria in Mali: a single-blind, dose-ranging, adaptive randomised phase 2 trial. *Lancet Infect. Dis.* 16 674–684. 10.1016/s1473-3099(15)00479-x 26906747 PMC10583596

[B9] Doumbia S. Chouong H. Traore S. F. Dolo G. Toure A. M. Coulibaly M. (2007). Establishing an insect disease vector functional genomics training center in Africa. *Afr. J. Med. Med. Sci.* 36(Suppl.) 31–33. 17703561

[B10] Doumbia S. O. Ndiaye D. Koita O. A. Diakite M. Nwakanma D. Coulibaly M. (2012). Improving malaria control in West Africa: interruption of transmission as a paradigm shift. *Acta Trop.* 121 175–183. 10.1016/j.actatropica.2011.11.009 22142790 PMC3294075

[B11] Doumbo O. K. Krogstad D. J. (1998). Doctoral training of African scientists. *Am. J. Trop. Med. Hyg.* 58 127–132. 10.4269/ajtmh.1998.58.127 9502592

[B12] Fairhurst R. M. Nayyar G. M. Breman J. G. Hallett R. Vennerstrom J. L. Duong S. (2012). Artemisinin-resistant malaria: research challenges, opportunities, and public health implications. *Am. J. Trop. Med. Hyg.* 87 231–241. 10.4269/ajtmh.2012.12-0025 22855752 PMC3414557

[B13] Flegg J. A. Guerin P. J. White N. J. Stepniewska K. (2011). Standardizing the measurement of parasite clearance in falciparum malaria: the parasite clearance estimator. *Malar. J.* 10 339. 10.1186/1475-2875-10-339 22074219 PMC3305913

[B14] Foundations for the National Institutes of Health (2018). *Biomarkers Consortium.* Available at: https://fnih.org/what-we-do/biomarkers-consortium/about/resources (accessed October 4 2018).

[B15] Gezmu M. DeGruttola V. Dixon D. Essex M. Halloran E. Hogan J. (2011). Strengthening biostatistics resources in sub-Saharan Africa: research collaborations through U.S. partnerships. *Stat. Med.* 30 695–708. 10.1002/sim.4144 21394746 PMC4562470

[B16] Gurwitz K. T. Aron S. Panji S. Maslamoney S. Fernandes P. L. Judge D. P. (2017). Designing a course model for distance-based online bioinformatics training in Africa: the H3ABioNet experience. *PLoS Comput. Biol.* 13:e1005715. 10.1371/journal.pcbi.1005715 28981516 PMC5628786

[B17] Gutierrez J. B. Harb O. S. Zheng J. Tisch D. J. Charlebois E. D. Stoeckert C. J. (2015). A framework for global collaborative data management for malaria research. *Am. J. Trop. Med. Hyg.* 93 (3 Suppl) 124–132. 10.4269/ajtmh.15-0003 26259944 PMC4574270

[B18] Harvard T.H. Chan School of Public Health (2018). *Fogarty Global Health Training Program, University of Sciences, Techniques and Technologies of Bamako, Mali [Online].* Available at: https://sites.sph.harvard.edu/global-health-research-partnership/sites/university-of-sciences-techniques-and-technologies-of-bamako-mali/ (accessed July 17 2018).

[B19] Hayashi C. (1998). *“What is Data Science? Fundamental Concepts and a Heuristic Example”.* Tokyo: Springer 40–51. 10.1007/978-4-431-65950-1_3

[B20] Head M. G. Goss S. Gelister Y. Alegana V. Brown R. J. Clarke S. C. (2017). Global funding trends for malaria research in sub-Saharan Africa: a systematic analysis. *Lancet Glob. Health* 5 e772–e781. 10.1016/s2214-109x(17)30245-0 28668230 PMC5567191

[B21] Human Heredity and Health in Africa (2013). *H3Africa: Human Heredity & Health in Africa [Online].* Available at: https://h3africa.org/ (accessed October 17 2018).

[B22] Human Heredity and Health in Africa (2018). *H3Africa: Human Heredity and Health in Africa [Online].* Available: www.h3africa.org (accessed July 17 2018).

[B23] Human Heredity and Health in Africa (2019). *West African Center of Excellence for Global Health Bioinformatics Research Training [Online].* Available at: https://h3africa.org/index.php/consortium/projects/west-african-center-of-excellence-for-global-health-bioinformatics-research-training/ (accessed February 25 2019).

[B24] International Centre of Insect Physiology and Ecology (2018). *Eastern Africa Network of Bioinformatics Training (EANBiT) Training Course [Online].* Available at: http://cbid.icipe.org/apps/eanbit/ (accessed July 17 2018).

[B25] Jennings S. F. Ptitsyn A. A. Wilkins D. Bruhn R. E. Slikker W. Jr. (2004). Regional societies: fostering competitive research through virtual infrastructures. *PLoS Biol.* 2:e372. 10.1371/journal.pbio.0020372 15597114 PMC535569

[B26] Karikari T. K. (2015). Bioinformatics in Africa: the Rise of Ghana? *PLoS Comput. Biol.* 11:e1004308. 10.1371/journal.pcbi.1004308 26378921 PMC4574930

[B27] Karikari T. K. Quansah E. Mohamed W. M. (2015a). Developing expertise in bioinformatics for biomedical research in Africa. *Appl. Transl. Genom.* 6 31–34. 10.1016/j.atg.2015.10.002 26767162 PMC4699396

[B28] Karikari T. K. Quansah E. Mohamed W. M. (2015b). Widening participation would be key in enhancing bioinformatics and genomics research in Africa. *Appl. Transl. Genom.* 6 35–41. 10.1016/j.atg.2015.09.001 26767163 PMC4699381

[B29] Kirigia J. M. Wambebe C. (2006). Status of national health research systems in ten countries of the WHO African Region. *BMC Health Serv. Res.* 6:135. 10.1186/1472-6963-6-135 17052326 PMC1622748

[B30] Koita O. A. Murphy R. L. Fongoro S. Diallo B. Doumbia S. O. Traore M. (2016). Clinical research and the training of host country investigators: essential health priorities for disease-endemic regions. *Am. J. Trop. Med. Hyg.* 94 253–257. 10.4269/ajtmh.15-0366 26598570 PMC4751934

[B31] Koita O. A. Sangare L. Miller H. D. Sissako A. Coulibaly M. Thompson T. A. (2017). AQ-13, an investigational antimalarial, versus artemether plus lumefantrine for the treatment of uncomplicated Plasmodium falciparum malaria: a randomised, phase 2, non-inferiority clinical trial. *Lancet Infect. Dis.* 17 1266–1275. 10.1016/s1473-3099(17)30365-1 28916443 PMC5700806

[B32] Landoure G. Samassekou O. Traore M. Meilleur K. G. Guinto C. O. Burnett B. G. (2016). Genetics and genomic medicine in Mali: challenges and future perspectives. *Mol. Genet. Genomic Med.* 4 126–134. 10.1002/mgg3.212 27066513 PMC4799869

[B33] Lansang M. A. Dennis R. (2004). Building capacity in health research in the developing world. *Bull. World Health Organ.* 82 764–770. 15643798 PMC2623028

[B34] Maiga A. W. Fofana B. Sagara I. Dembele D. Dara A. Traore O. B. (2012). No evidence of delayed parasite clearance after oral artesunate treatment of uncomplicated falciparum malaria in Mali. *Am. J. Trop. Med. Hyg.* 87 23–28. 10.4269/ajtmh.2012.12-0058 22764287 PMC3391052

[B35] MEASURE Evaluation (2014). *Situation Analysis of Mali’s Health Information System [Online].* Available at: https://www.measureevaluation.org/resources/publications/sr-14-112-fr (accessed July 17 2018).

[B36] MEASURE Evaluation (2016). *Aligning Stakeholders for Health Information Systems Strengthening: One Step at a Time [Online].* Available at: https://www.measureevaluation.org/resources/publications/fs-16-168-en (accessed July 17 2018).

[B37] Miiro G. M. Oukem-Boyer O. O. Sarr O. Rahmani M. Ntoumi F. Dheda K. (2013). EDCTP regional networks of excellence: initial merits for planned clinical trials in Africa. *BMC Public Health* 13:258. 10.1186/1471-2458-13-258 23517572 PMC3623728

[B38] Mlotshwa B. C. Mwesigwa S. Mboowa G. Williams L. Retshabile G. Kekitiinwa A. (2017). The collaborative African genomics network training program: a trainee perspective on training the next generation of African scientists. *Genet. Med.* 19 826–833. 10.1038/gim.2016.177 28383545 PMC5509501

[B39] Mulder N. Abimiku A. Adebamowo S. N. de Vries J. Matimba A. Olowoyo P. (2018a). H3Africa: current perspectives. *Pharmgenomics Pers. Med.* 11 59–66. 10.2147/pgpm.S141546 29692621 PMC5903476

[B40] Mulder N. Schwartz R. Brazas M. D. Brooksbank C. Gaeta B. Morgan S. L. (2018b). The development and application of bioinformatics core competencies to improve bioinformatics training and education. *PLoS Comput. Biol.* 14:e1005772. 10.1371/journal.pcbi.1005772 29390004 PMC5794068

[B41] Mulder N. J. Adebiyi E. Adebiyi M. Adeyemi S. Ahmed A. Ahmed R. (2017). Development of bioinformatics infrastructure for genomics research. *Glob. Heart* 12 91–98. 10.1016/j.gheart.2017.01.005 28302555 PMC5582980

[B42] Mulder N. J. Adebiyi E. Alami R. Benkahla A. Brandful J. Doumbia S. (2016). H3ABioNet, a sustainable pan-African bioinformatics network for human heredity and health in Africa. *Genome Res.* 26 271–277. 10.1101/gr.196295.115 26627985 PMC4728379

[B43] Mwangoka G. Ogutu B. Msambichaka B. Mzee T. Salim N. Kafuruki S. (2013). Experience and challenges from clinical trials with malaria vaccines in Africa. *Malar. J.* 12:86. 10.1186/1475-2875-12-86 23496910 PMC3599886

[B44] National Institute of Allergy and Infectious Diseases (2018a). *ICEMR Program Overview [Online].* Available at: https://www.niaid.nih.gov/research/icemr-program-overview (accessed November 2 2018).

[B45] National Institute of Allergy and Infectious Diseases (2018b). *West Africa International Center of Excellence for Malaria Research, [Online].* Available at: https://www.niaid.nih.gov/research/west-africa-icemr (accessed August 22 2018).

[B46] National Institutes of Health (2018). *H3ABionet: Pan African Bioinformatics Network for H3Africa [Online].* Available at: www.h3abionet.org (accessed July 17 2018).

[B47] Ndabarora E. Chipps J. A. Uys L. (2014). Systematic review of health data quality management and best practices at community and district levels in LMIC. *Inform. Dev.* 30 103–120. 10.1177/0266666913477430

[B48] Niare K. Dara A. Sagara I. Sissoko M. S. Guindo C. O. Cisse N. H. (2016). In vivo efficacy and parasite clearance of artesunate + sulfadoxine-pyrimethamine versus artemether-lumefantrine in mali. *Am. J. Trop. Med. Hyg.* 94 634–639. 10.4269/ajtmh.15-0503 26811430 PMC4775901

[B49] Nielsen M. W. Alegria S. Borjeson L. Etzkowitz H. Falk-Krzesinski H. J. Joshi A. (2017). Opinion: Gender diversity leads to better science. *Proc Natl. Acad. Sci. U.S.A.* 114 1740–1742. 10.1073/pnas.1700616114 28228604 PMC5338420

[B50] Oboh M. A. Singh U. S. Antony H. A. Ndiaye D. Badiane A. S. Ali N. A. (2018). Molecular epidemiology and evolution of drug-resistant genes in the malaria parasite Plasmodium falciparum in southwestern Nigeria. *Infect. Genet. Evol.* 66 222–228. 10.1016/j.meegid.2018.10.007 30316883

[B51] NEPAD Planning and Coordinating Agency (2010). *African Innovation Outlook 2010.* Pretoria: NEPAD Planning and Coordinating Agency

[B52] Portugal S. Tran T. M. Ongoiba A. Bathily A. Li S. Doumbo S. (2017). Treatment of chronic asymptomatic plasmodium falciparum infection does not increase the risk of clinical malaria upon reinfection. *Clin. Infect. Dis.* 64 645–653. 10.1093/cid/ciw849 28362910 PMC6075513

[B53] President’s Malaria Initiative (2018). *Mali Country Profile.* Available at: https://www.pmi.gov/docs/default-source/default-document-library/country-profiles/mali_profile.pdf?sfvrsn=24 (accessed October 4 2018).

[B54] Quansah E. McGregor N. W. (2018). Towards diversity in genomics: The emergence of neurogenomics in Africa? *Genomics* 110 1–9. 10.1016/j.ygeno.2017.07.009 28774809

[B55] Richie T. L. Billingsley P. F. Sim B. K. James E. R. Chakravarty S. Epstein J. E. (2015). Progress with Plasmodium falciparum sporozoite (PfSPZ)-based malaria vaccines. *Vaccine* 33 7452–7461. 10.1016/j.vaccine.2015.09.096 26469720 PMC5077156

[B56] Rotimi C. Abayomi A. Abimiku A. Adabayeri V. M. Adebamowo C. Adebiyi E. (2014). Research capacity. Enabling the genomic revolution in Africa. *Science* 344 1346–1348. 10.1126/science.1251546 24948725 PMC4138491

[B57] Sagara I. Piarroux R. Djimde A. Giorgi R. Kayentao K. Doumbo O. K. (2014). Delayed anemia assessment in patients treated with oral artemisinin derivatives for uncomplicated malaria: a pooled analysis of clinical trials data from Mali. *Malar. J.* 13:358. 10.1186/1475-2875-13-358 25217396 PMC4177171

[B58] SAMRC (2018). *Genomics centre in Cape Town to Decode Genes [Online].* Available at: http://www.mrc.ac.za/media-release/genomics-centre-cape-town-decode-genes (accessed February 23 2019).

[B59] Schieffelin J. S. Shaffer J. G. Goba A. Gbakie M. Gire S. K. Colubri A. (2014). Clinical illness and outcomes in patients with Ebola in Sierra Leone. *N. Engl. J. Med.* 371 2092–2100. 10.1056/NEJMoa1411680 25353969 PMC4318555

[B60] Schoonbaert D. (2009). PubMed growth patterns and visibility of journals of Sub-Saharan African origin. *J. Med. Libr. Assoc.* 97 241–243; author reply 243. 10.3163/1536-5050.97.4.004 19851485 PMC2759164

[B61] SciDevNet (2004). *African Bioinformatics Network [Online].* Available at: https://www.scidev.net/global/health/link/african-bioinformatics-network.html (accessed February 25 2019).

[B62] Science Blog (2000). *National Institutes of Health Press Release: New Malaria Research Facility Dedicated in Mali, West Africa [Online].* Available at: http://www3.scienceblog.com/community/older/archives/B/nih1653.html (accessed October 4 2018).

[B63] Shaffer J. G. Doumbia S. O. Ndiaye D. Diarra A. Gomis J. F. Nwakanma D. (2018). Development of a data collection and management system in West Africa: challenges and sustainability. *Infect Dis. Poverty* 7:125. 10.1186/s40249-018-0494-4 30541626 PMC6292095

[B64] Shaffer J. G. Grant D. S. Schieffelin J. S. Boisen M. L. Goba A. Hartnett J. N. (2014). Lassa fever in post-conflict sierra leone. *PLoS Negl. Trop. Dis.* 8:e2748. 10.1371/journal.pntd.0002748 24651047 PMC3961205

[B65] Sissoko M. S. Sissoko K. Kamate B. Samake Y. Goita S. Dabo A. (2017). Temporal dynamic of malaria in a suburban area along the Niger River. *Malar. J.* 16:420. 10.1186/s12936-017-2068-5 29058578 PMC5651586

[B66] Takala-Harrison S. Laufer M. K. (2015). Antimalarial drug resistance in Africa: key lessons for the future. *Ann. N. Y. Acad. Sci.* 1342 62–67. 10.1111/nyas.12766 25891142 PMC4527866

[B67] Tastan Bishop O. Adebiyi E. F. Alzohairy A. M. Everett D. Ghedira K. Ghouila A. (2015). Bioinformatics education–perspectives and challenges out of Africa. *Brief Bioinform.* 16 355–364. 10.1093/bib/bbu022 24990350 PMC4364068

[B68] The Economist (2017). *Science Remains Male-Dominated.* Available at: https://www.economist.com/science-and-technology/2017/03/11/science-remains-male-dominated (accessed October 4 2018).

[B69] The World Bank (2019). *Research and development expenditure (% of GDP) [Online].* Available at: https://data.worldbank.org/indicator/gb.xpd.rsdv.gd.zs?year_high_desc=true (accessed February 25 2019).

[B70] Tishkoff S. A. Reed F. A. Friedlaender F. R. Ehret C. Ranciaro A. Froment A. (2009). The genetic structure and history of Africans and African Americans. *Science* 324 1035–1044. 10.1126/science.1172257 19407144 PMC2947357

[B71] Traore B. Oliveira F. Faye O. Dicko A. Coulibaly C. A. Sissoko I. M. (2016). Prevalence of Cutaneous Leishmaniasis in Districts of High and Low Endemicity in Mali. *PLoS Negl. Trop. Dis.* 10:e0005141. 10.1371/journal.pntd.0005141 27898671 PMC5127506

[B72] U.S. Department of Health and Human Services (2018). *NIH RePORTER [Online].* Available at: https://projectreporter.nih.gov/reporter.cfm (accessed November 14 2018).

[B73] U.S. Department of State (2019). *Exchange Visitor Program [Online].* Available at: https://j1visa.state.gov/participants/how-to-apply/eligibility-and-fees/ (accessed February 27 2019).

[B74] United States Central Intelligence Agency (2018). *The World Factbook [Online].* Available at: https://www.cia.gov/library/publications/the-world-factbook/rankorder/2119rank.html (accessed November 14 2018).

[B75] Whitworth J. Sewankambo N. K. Snewin V. A. (2010). Improving implementation: building research capacity in maternal, neonatal, and child health in Africa. *PLoS Med.* 7:e1000299. 10.1371/journal.pmed.1000299 20625547 PMC2897765

[B76] Wikipedia (2019a). *Bioinformatics [Online].* Available at: https://en.wikipedia.org/wiki/Bioinformatics (accessed February 26 2019).

[B77] Wikipedia (2019b). *Languages of Mali [Online].* Available at: https://en.wikipedia.org/wiki/Languages_of_Mali (accessed February 26 2019).

[B78] Woolley A. W. Chabris C. F. Pentland A. Hashmi N. Malone T. W. (2010). Evidence for a collective intelligence factor in the performance of human groups. *Science* 330 686–688. 10.1126/science.1193147 20929725

[B79] World Health Organization (2004). *Developing Health Management Information Systems: A Practical Guide for Developing Countries.* Available at: http://www.wpro.who.int/health_services/documents/developing_health_management_information_systems.pdf (accessed October 4 2018).

[B80] World Health Organization (2015). *Africa’s Women in Science [Online].* Available at: www.who.int/tdr/research/gender/Women_overview_piece.pdf (accessed October 4 2018).

